# Serum levels of selected cytokines [interleukin (IL)-17A, IL-18, IL-23] and chemokines (RANTES, IP10) in the acute phase of immunoglobulin A vasculitis in children

**DOI:** 10.1007/s00296-019-04415-4

**Published:** 2019-08-29

**Authors:** Majka Jaszczura, Elżbieta Mizgała-Izworska, Elżbieta Świętochowska, Edyta Machura

**Affiliations:** 1 Department of Paediatrics, School of Medicine with the Division of Dentistry in Zabrze, Medical University of Silesia in Katowice, ul. 3-go Maja 13-15, 41-800 Zabrze, Poland; 2 Department of Family Medicine, School of Medicine with the Division of Dentistry in Zabrze, Medical University of Silesia in Katowice, Zabrze, Poland; 3 Department of Medical and Molecular Biology, School of Medicine with the Division of Dentistry in Zabrze, Medical University of Silesia in Katowice, Zabrze, Poland

**Keywords:** Henoch–Schonlein purpura, IgAV, Interleukin 17, Interleukins, Chemokines

## Abstract

The pathogenesis of the immunoglobulin A vasculitis (IgAV) is still unknown. The available data shows that interleukin (IL)-17, IL-18, IL-23, regulated on activation, normal T cell expressed and secreted (CCL 5, RANTES), and interferon (IFN)-γ-inducible protein 10 (IP10) participate in the pathogenesis of IgAV by influencing the recruitment of leukocytes to the site of inflammation. The aim of this study was to analyze the serum concentration of IL-17A, IL-18, IL-23, RANTES, and IP10 in patients with acute IgAV compared to healthy children. Moreover, we wanted to assess the suitability of the levels of tested cytokines to predict the severity of the disease. All children with IgAV hospitalized in our institution between 2012 and 2017 were included in the study. Cytokines levels were determined in a serum sample secured at admission to the hospital. Basic laboratory tests have also been analyzed. IL-17A, IL-18, and IL-23 were significantly higher in whole IgAV group (52.25 pg/ml; 164.1 pg/ml and 700 pg/ml, respectively) than in the control group (27.92 pg/ml; 140.1 pg/ml and 581.5 pg/ml, respectively). The receiver operating characteristic (ROC) curve analysis revealed the largest area under the curve (AUC 0.979, *p* < 0.001) for the IL-17A with 95.1% sensitivity and 91.7% specificity. There were no significant differences in cytokine levels depending on the severity of the IgAV. Although the serum levels of the IL-17A, IL-18, and IL-23 increase significantly in the acute phase of the IgAV, they cannot be used as indicators of predicting the course of the disease. IL-17A seems to be a good predictor of IgAV occurrences.

## Introduction

Immunoglobulin A vasculitis (IgAV), formerly known as Henoch–Schonlein purpura, is the most common systemic vasculitis in childhood with a reported incidence of 3–26.7/100 000 [1]. The pathogenesis of the IgAV still remains unclear. In recent years, the importance of the autoimmune hypothesis of the pathogenesis of the disease increased significantly [2, 3]. Taking new reports into account, the view on the pathogenesis of autoimmune diseases has changed, and the Th1 lymphocytes (Th1)/Th2 lymphocytes (Th2) paradigm is in decline. Th1, which produce IFN-γ and IL-2, enhance cell-mediated immune response, activate macrophages and cytotoxic T lymphocytes, and stimulate the production of complement-activating IgG1 and IgG3 antibodies. Th2 stimulate mainly the humoral immune response, the production of IgA, IgE, IgG4 antibodies, as well as the growth and differentiation of mast cells and eosinophils. Consequently, it is responsible for the development of allergic reactions. Initially, it was believed that the development of autoimmune diseases with the dominant cell type response (multiple sclerosis, type I diabetes, rheumatoid arthritis) was mainly due to Th1, whereas Th2 was responsible for the development of diseases caused mainly by antibodies (myasthenia gravis, pemphigus). The hypothesis saying that Th1 cells, which produce IFN-γ, gives rise to the number of autoimmune diseases lost its importance when the research conducted on animal models of multiple sclerosis proved that IFN-γ-deficient mice are more susceptible to disease [4, 5]. In addition, the view on the pathogenesis of autoimmune diseases has changed at the moment of discovering lymphocytes subpopulation, which secretory profile differ significantly from Th1 and Th2. These lymphocytes were called Th17 lymphocytes (Th17) because they secreting mainly IL-17 [6, 7]. The development and maturation of Th17 are synergistically stimulated by IL-18 and IL-23.

IL-18 is a member of the IL-1 superfamily and, similarly to IL-1β, it is synthesized as an inactive precursor. IL-18 is a proinflammatory and immunoregulatory cytokine, with IL-12 or IL-15, it stimulates the production of IFNγ by Th1 cells. However, in the absence of IL-12 and IL-15, IL-18 plays a valid role in Th2 differentiation [8, 9]. The role of IL-18 in IgAV is connected with IL-23. These interleukins synergistically induce γδT cells to produce IL-17 [8, 10]. It is believed that IL-23, which is essential for proliferation, terminal differentiation and the sustained production of IL-17, increases the pathogenicity of Th17 cells. IL-23 also makes Th17 cells to reduce the expression of IL-27 receptors, which are suppression mediators [11–13].

IL-17 is a cytokine described and named in 1995. Presently, there are known six homologues molecules of IL-17 (IL-17A to IL-17F) [12]. IL-17A is a proinflammatory cytokine with many biological functions, including upregulating proinflammatory genes expressions. It induces the production of IL-6, IL-8, TNF, chemokines, matrix metalloproteinases in a variety of cells (fibroblasts, epithelial and endothelial cells, macrophages, dendritic cells, chondrocytes, and osteoblasts), and plays a protective role in mucosal immunity to extracellular bacteria and fungi. Several types of immune cells, for instance, γδ T cells, natural killer cells (NK), invariant NKT cells, Th17 cells, neutrophils, and mast cells, produce IL-17A [14]. The contribution of IL-17A to IgAV pathogenesis may be due to its effect on neutrophils migration.

Another important factors in the process of activation, adhesion, and the recruitment of leukocytes to the place of inflammation are chemokines such as RANTES or IP10. Considering the perivascular accumulation of neutrophils in the histopathological result in IgAV, chemokines can be valid factors in the process of damaging the tissue during vasculitis [15].

The aim of this study was to analyze the serum concentration of IL-17A, IL-18, IL-23, RANTES, and IP10 in patients with acute IgAV compared to healthy children. Moreover, we wanted to assess the suitability of the levels of tested cytokines to predict the severity of the disease.

## Materials and methods

Seventy-one patients with IgAV hospitalized in the Paediatric Department of the Clinical Hospital No. 1 in Zabrze in 2012–2017, who met the EULAR/PRINTO/PRES diagnostic criteria [16], were included in the study. Nine patients were excluded from the current analysis due to the lack of serum samples. Finally, there were 62 patients who were examined.

Based on a medical history and the clinical scoring scale (Table 1) from Muslu A et al. and De Matia et al. modified by Fessatou et al. the severity of the disease was assessed (mild ≤ 4 points, severe > 4). This scale assesses the severity of joint, renal and gastrointestinal symptoms [17–19]. The presence of systemic involvement was defined as the occurrence of GT bleeding and/or kidneys involvement. Gastrointestinal bleeding was defined as haematemesis, melaena, hematochezia, and the positive faecal occult blood test (FOBT). The renal involvement was defined as hematuria (> 5 RBC in the field of vision), macroscopic hematuria or proteinuria (> 300 mg/24 h). We made two different IgAV patients divisions. In the first, the entire group was divided into two subgroups depending on the severity score. In the second the whole group was divided into two subgroups depending on systemic involvement.

**Table 1:** The clinical scoring system in patients with immunoglobulin A vasculitis [17–19]

Arthritis score	
0	No symptom
1	Artralgia and/or slight swelling (normal walk)
2	Artralgia and/or moderate swelling (difficult to walk)
3	Artralgia and/or severe swelling (refuse to walk)
Abdominal score	
0	No symptom
1	Mild abdominal pain and/or occult blood in stool (+)
2	Moderate abdominal pain (transient complaints brought to medical attention) and/or occult blood in stool (++/+++)
3	Severe abdominal pain and/or melena and/or hematemesis and/or intussusception and/or surgical consultation required
Renal score	
0	No proteinuria and/or 3–5 RBC/HPF
1	Proteinuria 30 mg/dl and/or microalbuminuria and/or 10–15 RBC/HPF
2	Proteinuria 30–150 mg/dl and/or > 50 RBC/HPF
3	Proteinuria 150 mg/dl and/or macroscopic haematuria

*RBC* red blood cells, *HPF* high-power field

The control group consisted of 43 healthy children, selected in terms of age and gender (Table 2). Children from the control group attended the outpatient pediatric clinic for non-immunological and non-inflammatory health problems, and they needed venous puncture.

**Table 2:** Demographic, clinical and selected laboratory characteristic of immunoglobulin A vasculitis patients compared to the control group

Parameter	Immunoglobulin A vasculitis group (*n* = 62)	Control group (*n* = 43)
Median age (years) (*Q*_25_–*Q*_75_)	6 (5–9)	7 (4–13)
Gender M/F	30/32	20/28
BMI (kg/m^2^)	16.17 (14.5–19)	16.9 (15.7–18.9)
Median laboratory data (*Q*_25_–*Q*_75_)		
Hemoglobin (g/dL)	12.9 (12.5–14)	12.94 (12.13–13.7)
Platelets (× 10^3^/µl)	353 (289–399)*	303 (243–333)
White blood cell (× 10^3^/µl)	10.15 (8–13)*	6.6 (5.58–7.94)
Neutrophil (× 10^3^/µl)	6.1 (4.5–8.8)*	3.47 (2.31–4.16)
Lymphocyte (× 10^3^/µl)	2.86 (2.2–4.1)	2.4 (1.93–3.14)
C-reactive protein (mg/l)	7.4 (2.98–21.99)*	0.6 (0.24–0.98)
Neutrophil to lymphocyte ratio (NLR)	1.93 (1.29–3.12)*	1.22 (0.96–1.94)
Platelet to lymphocyte ratio (PLR)	120.67 (88.8–147.9)	126.79 (95.28–152.88)
IgA (g/l)	1.77 (1.39–2.17)*	1.37 (0.78–1.9)
IgG (g/l)	9.37 (7.77–12.24)	10.25 (8.49–10.96)
IgM (g/l)	0.89 (0.74–1.18)	0.98 (0.78–1.23)
C3 (g/l)	1.2 (1.13–1.39)	Not studied
C4 (g/l)	0.24 (0.2–0.28)	Not studied
Median duration of symptoms on admission (days) (*Q*_25_–*Q*_75_)	3 (2–5)	–
Month of diagnosis		
Spring	14 (23%)	Not applicable (NA)
Summer	11 (18%)	
Autumn	20 (32%)	
Winter	17 (27%)	
Trigger		
Respiratory tract infection	28 (45%)	NA
Gastrointestinal infection	3 (5%)	
Unknown	31 (50%)	
Symptoms and signs		
Purpura (P)	62 (100%)	NA
Arthritis/arthralgia (A)	38 (61%)	
Gastointestinal tract involvment (GI)	39 (63%)	
Abdominal pain	35 (56%)	
Gastointestinal bleeding (GB)	32 (52%)	
Melena/hematochezia	5 (8%)	
Positive FOBT	31 (50%)	
Intussusception	1 (1.6%)	
Obstruction of the GT susp.	2 (3.2%)	
Appendictitis susp.	1 (1.6%)	
Kidneys involvement (KI)	15 (24%)	
Proteinuria (> 300 mg/24 h)	1 (1.6%)	
Hematuria (> 5 RBC/HPF)	4 (6.5%)	
Proteinuria and hematuria	10 (16%)	
Other		
Scrotum involvment (epididymitis. oedema)	2 (3.2%)	
Headache	1 (1.6%)	
Severity score		
Mild (≤ 4 points)	51 (82%)	NA
Severe (> 4 points)	11 (18%)	
Systemic involvment		
Yes: gastointestinal bleeding (GB) + kidneys involvement (KI)	34 (66%)	NA
No: purpura (P) + arthritis/arthralgia (A)	28 (34%)	

*FOBT* faecal occult blood test, *RBC* red blood cells, *HPF* high-power field, *IgAV* immunoglobulin A vasculitis

*p < 0.05 IgAV group in comparison with control group

At the time of the patient’s admission to the hospital (before the start of treatment), a venous blood sample (standard EDTA tubes) for laboratory tests was collected. Basic laboratory tests were performed within the first hour after admission. The following laboratory data were recorded: hemoglobin level (Hgb), white blood cell count (WBC), neutrophil and lymphocyte count, platelet count (PLT), C-reactive protein (CRP), and immunoglobulins level. The haemogram-derived parameters were determined by the use of an automatic hematology analyzer and they were used to calculate neutrophil to lymphocyte ratio (NLR), and platelet to lymphocyte ratio (PLR).

Serum samples were frozen and stored at − 40 °C until the levels of cytokines were detected. A commercial enzyme-linked immunosorbent assay (ELISA) kits were used to measure the levels of cytokines. The IL-17A, IL-23 and IP10 concentrations were measured by the use of Diaclone (France) kits. The sensitivity was 2.3 pg/ml; < 20 pg/ml and 5.7 pg/ml, respectively. The IL-18 and RANTES levels were detected by the use of Cloud-Clone Corp. (USA) kits (with 5.6 pg/ml and 0.061 ng/ml sensitivity, respectively). Absorbance readings were made using the μQuant (BioTek, USA), while the results were processed using the KCJunior (BioTek, USA). All analytical procedures were in accordance with the manufacturer’s recommendations attached to the kits.

The presented study was approved by the Ethics Committee of the Medical University of Silesia in Katowice on 01.07.2014 (KNW/0022/KB1/66/14; KNW/0022/KB1/66/III/14/16/17) and written informed consent was obtained from children’s parents.

### Statistical evaluation

Statistical calculations were made using the Statistica 13.0 (StatSoft, Poland). To determine the distribution of analyzed variables, Shappiro Wilk test was performed. Variables with a non-normal distribution were presented as median with an interquartile range while variables with a normal distribution were presented as a mean with standard deviation. A comparative analysis of groups was performed using the Mann–Whitney *U* test. Univariate logistic regression analysis and receiver operating characteristic (ROC) analysis were performed to determine the usefulness of analyzed variables as potential biomarkers. Youden index method was used to determine cut-off points. *p* values < 0.05 were considered statistically significant.

## Results

The children from the study and control groups did not differ significantly in terms of age, sex, and BMI. The demographic and clinical characteristics of both groups are presented in Table 2.

The median age of IgAV children was 6 years. Patients were included after a median duration of symptoms of 3 days. The majority of cases (59%) were diagnosed in autumn and winter, and in 49% of patients, the respiratory tract infection was a trigger. All patients showed palpable purpura, especially on lower extremities. In the case of some children, the rash was preceded by arthritis (9.7%) or gastrointestinal symptoms (11.3%). In the course of the disease, gastrointestinal symptoms were observed in 39 children (63%) and the glomerulonephritis symptoms in 15 (24%). Depending on the severity scale of the disease, 82% of patients (*n* = 51) were classified as mild and 18% (*n* = 11) as severe. Depending on the systemic involvement, 66% (*n* = 34) of patients revealed signs of systemic involvement, including GT bleeding and/or glomerulonephritis. 34% (*n* = 28) of patients were non-systemic and they showed skin and joint symptoms only. Isolated renal involvement was observed in 2 patients. Children with IgAV had significantly higher values of WBC, neutrophils, PLT, NLR, CRP and IgA compared to the control group (Table 2).

The whole IgAV group had statistically significantly higher values of IL-17A, IL-18, and IL-23 in comparison to the control group (*p* < 0.001). Similarly, in all separate subgroups, the IL-17A, IL-18, and IL-23 levels were significantly higher in comparison to the control group (mild vs control, severe vs control, systemic involvement vs control, non-systemic involvement vs control: *p* < 0.05). There were no significant statistical differences in the values of RANTES and IP10 between separate subgroups and the control group.

We did not show any significant differences of serum IL-17A, IL-18, IL-23, RANTES, and IP10 levels between the mild and severe group (*p* = 0.052; *p* = 0.33; *p* = 0.69; *p* = 0.35; *p* = 0.44, respectively) and between systemic involvement group and without-systemic involvement group (*p* = 0.93; *p* = 0.97; *p* = 0.73; *p* = 0.53; *p* = 0.87, respectively). The comparison of cytokines levels in all separate subgroups and in the control group is presented in Table 3.

**Table 3:** Comparison of selected cytokines and chemokines levels between all separated immunoglobulin A vasculitis subgroups and the control group

Parameter	Immunoglobulin A vasculitis median (*Q*_25_–*Q*_75_)	Control group (*n* = 43) median (*Q*_25_–*Q*_75_)
IL-17A (pg/ml)		
All IgAV group (*n* = 62)	52.25 (43.2–70.2)*	27.92 (22.5–32.3)
Severe course (*n* = 11)	43.2 (40.2–52.7)*	
Mild course (*n* = 51)	55.8 (46.5–70.7)*	
Systemic involvment (*n* = 34)	52.25 (43.2–70.2)*	
Without systemic involvment (*n* = 28)	54.75 (44.15–70.1)*	
IL-18 (pg/ml)		
All IgAV group (*n* = 62)	164.1 (150.1–185.3)*	140.1 (126.6–151.2)
Severe course (*n* = 11)	155.7 (139.5–189.3)*	
Mild course (*n* = 51)	165.3 (151.2–184.6)*	
Systemic involvment (*n* = 34)	163.6 (149.8–188.6)*	
Without systemic involvment (*n* = 28)	164.75 (152.4–180.55)*	
IL-23 (pg/ml)		
All IgAV group (*n* = 62)	700 (549–804)*	581.5 (513.5–644.5)
Severe course (*n* = 11)	735 (489–839)*	
Mild course (*n* = 51)	698 (549–802)*	
Systemic involvment (*n* = 34)	668 (571–804)*	
Without systemic involvment (*n* = 28)	711.5 (549–825.5)*	
RANTES/CCL5 (pg/ml)		
All IgAV group (*n* = 62)	5465.5 (4789–7045)	5777 (4897.5–6218)
Severe course (*n* = 11)	5583 (5134–7281)	
Mild course (*n* = 51)	5348 (4713–7045)	
Systemic involvment (*n* = 34)	5784 (4927–7045)	
Without systemic involvment (*n* = 28)	5083 (4500–7161.5)	
IP10/CXCL10 (pg/ml)		
All IgAV group (*n* = 62)	75.35 (60.8–89.4)	73.8 (63.6–85.6)
Severe course (*n* = 11)	71.6 (58.9–83.6)	
Mild course (*n* = 51)	76.2 (61.2–89.6)	
Systemic involvment (*n* = 34)	75.1 (60.3–88.9)	
Without systemic involvment (*n* = 28)	75.35 (62.5–89.5)	

*IgAV* immunoglobulin A vasculitis, *RANTES* regulated on activation, normal T-cell expressed and secreted, *IP10* interferon (IFN)-γ-inducible protein 10

*p < 0.05 in comparison with control group

The univariate logistic regression analysis to identify potential biomarkers of IgAV, showed that IgAV was associated with higher IL-17A, IL-18, and IL-23 levels. The indicator with the highest odds ratio (OR 1.434, *p* < 0.001) is IL-17A (Table 4).

**Table 4:** Logistic regression analysis of the association of IL-17A, IL-18, IL-23, RANTES, IP-10 and immunoglobulin A vasculitis

Parameter	OR	95% CI lower	95% CI upper	*p* value
IL-17A	1.434	1.214	1.693	**< 0.001**
IL-18	1.058	1.032	1.084	**< 0.001**
IL-23	1.006	1.003	1.01	**< 0.001**
RANTES/CCL5	1	1	1.001	0.162
IP-10/CXCL10	1.011	0.985	1.037	0.426

Bold values are statistically significant

*OR* odds ratio, *95% CI* lower and upper limits of the 95% confidence interval for the odds ratio, *RANTES* regulated on activation, normal T-cell expressed and secreted, *IP10* interferon (IFN)-γ-inducible protein 10

**Fig. 1:**
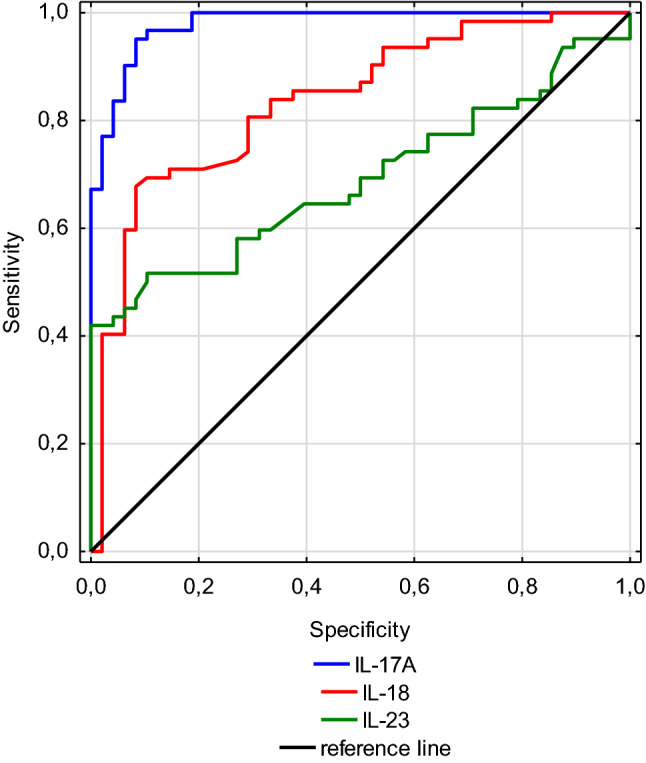
Comparison of receiver operating characteristic (ROC) curves of IL-17A, Il-18, IL-23 for immunoglobulin A vasculitis predicting

ROC curve analysis revealed that statistically significant predictors of IgAV are IL-17A, IL-18 and, IL-23. The largest area under the curve (AUC) was demonstrated for the IL-17A (AUC = 0.979, *p* < 0.001). The optimal cut-off value of IL-17A level determined with the use of the Youden index at the level of 38.7 pg/ml, showed the highest sensitivity (95.1%), and specificity (91.7%). For IL-18 the AUC was 0.837 (with 67.7% sensitivity and 91.7% specificity, *p* < 0.001) with optimal cut-off value at 155.4 pg/ml. For IL-23 the AUC was 0.689 (with 51.6% sensitivity and 89.6% specificity, *p* < 0.001) with cut-off value at 698 pg/ml. The comparison of ROC curves for selected parameters is shown in Fig. 1.

## Discussion

In the present study, we showed significant differences in the levels of IL-17A, IL-18, and IL-23 between the groups studied. The significantly higher serum level of mentioned cytokines in children with acute IgAV may confirm their participation in the pathogenesis of the disease. We did not show any differences in the levels of IP-10 and RANTES, as well as we did not prove the relationship between the levels of tested cytokines and the course of the disease. Among tested cytokines, IL-17A seems to be a good predictor of IgAV occurrence. Most of the research conducted by other authors also showed an elevated concentration of IL-17 in the acute phase of the disease [20–22]. Li et al. and Jen et al. agree that the elevated level of IL-17 is not surprising because of the increased proportion of Th17 in acute IgAV. They also suggest that an aberrant activation of Th17 may play an important role in the development of vasculitis [20, 21]. These studies suggest that apart from the significant contribution of humoral immunity, participation in the pathogenesis of IgAV has also cellular immunity. Moreover, Xu et al. showed that the polymorphism of IL-17A rs2275913 is strongly associated with IgAV susceptibility but there is no relationship between IL-17F genes polymorphism and IgAV [23]. Only Audemard-Verger et al. did not show markedly elevated levels of Th17- related cytokines (IL-17A, IL-22, IL-23) in adults with IgAV [24]. So far, only one study [20] evaluated the relationship between IL-17 and clinical symptoms, similarly to our research, it did not show any significant dependence. The lack of such a relationship may result from the insufficient number of examined patients and the relatively mild course of the disease. It is also possible that despite a significant increase in IL-17 level in the acute phase of the IgAV, it has no effect on its course. Further studies are needed to resolve these doubts.

The crucial function of IL-17 is to attract neutrophils to the site of infection. Neutrophils are the first effector cells that flow into the site of inflammation, remove extracellular pathogens and affect the activation, regulation, and function of other immune cells [25]. IL-17A stimulates epithelial, endothelial and fibroblastic cells to produce granulocyte colony-stimulating factor (G-CSF). It may also promote the maturation of hematopoietic progenitor cells into neutrophils [26]. IL-17A stimulates the secretion of CXC chemokines (CXCL-1, CXCL-6, CXCL-8) and synergizes with other cytokines (IL-1, IL-2, TNF), which leads to the activation of neutrophils and their infiltration into tissues [12, 27]. In addition to the protective role of stimulating the production of many antibacterial proteins (b-defensins, mucins: MUC5AC, MUC5B, S100 calgranulins, lipocalin 2), IL-17 appears to be important in autoimmunity as evidenced by the experimental autoimmune encephalomyelitis (EAE) [12, 28]. Both in this and in previous studies, the number of neutrophils and of the NLR was significantly increased in IgAV children. When we consider the role of IL-17 in the recruitment of neutrophils, it confirms their joint participation in IgAV pathogenesis [29–31]. The NLR was also useful in predicting the occurrence of systemic involvement in IgAV, which was shown in our earlier analysis [31]. To determine if the increase in IL-17A level is specific for IgAV, its level should be compared with other diseases with inflammatory pathogenesis. According to the literature, the increase of IL-17A was observed in autoimmune diseases such as rheumatoid arthritis, multiple sclerosis, systemic lupus erythematosus, and inflammatory bowel disease [9, 12, 32].

The research conducted by other authors showed that children with IgAV had statistically higher proportions of Th2 and Th17 in peripheral blood mononuclear cells (PBMCs), while the proportions of Th1 did not differ [20, 21]. Th17 cells, in addition to IL-17A secretion, are the source of IL-10, IL-21, IL-22 and TNFα which, by affecting the production of numerous chemokines, can modulate a cellular immune response [33]. The Th17 differentiation process was divided into two stages. In the priming stage, the transforming growth factor (TGF) β and IL-6 are needed to early differentiation [34], while IL-23 plays a crucial role in the maturation stage. Contrary to the previously published studies, which did not show elevated IL-23 level in IgAV patients [20, 35], our research has proved its elevated level in acute IgAV patients. It is considered that pathogenetic phenotype of Th17 cells is maintained by IL-23, therefore, some researchers suggest that low IL-23 level can explain the mild and short course of the disease [20]. In our work, we did not show any relation between IL-23 and the severity of the disease. There has been shown the role of the IL-23/IL-17 axis in the pathogenesis of spondyloarthritis and psoriasis [14, 36]. IL-23 correlation with disease severity in rheumatoid arthritis has been also demonstrated [37]. In addition, IL-23 affects hematogenesis and stimulates the production of platelets and neutrophils, which may be related to the significantly higher number of PLT and neutrophils found in our study in IgAV patients.

As mentioned above, our work indicates an elevated level of IL-18 in acute IgAV patients. The results of previous studies are partly divergent. Mahajan et al. did not observe a difference in IL-18 level in IgAV patients and controls, but they noticed that IL-18 level was significantly higher in the acute phase than in the remission phase of the disease [38]. Wang et al. showed a significantly higher level of IL-18 and IL-18 binding protein (IL-18BP) in active IgAV. They also showed a higher level of IL-18 in the acute phase compared with remission [39]. The elevated concentration of IL-18, which affects Th17 maturation and Th2 differentiation, may cause the above-mentioned increased proportions of the Th17 and Th2 in IgAV patients. In addition, IL-18 stimulates IL-17 production by γδT cells [9].

Our study did not show significant differences in RANTES and IP10 concentration between the groups studied. However, it was shown that chemokines play a role in many inflammatory diseases such as systemic lupus erythematosus, Kawasaki disease, Wegener’s granulomatosis, and Behcet’s disease [40–42]. IP10 is a strong attractant for macrophages and NK cells. RANTES is responsible for the recruitment of T cells, dendritic cells, eosinophils, NK cells, and monocytes to sites of inflammation [42, 43]. Two previous studies have shown elevated levels of RANTES in acute IgAV, and one of them has also shown its increase in patients with internal organ involvement compared to patients without organ involvement [15, 44]. Yu et al. suggest the association of the RANTES gene polymorphism with an increased risk of IgAV nephritis [15]. IP10 research is very divergent. Each of the 4 available IP10 studies in acute IgAV provides different results of its increase [15, 24] or decrease [45]. Chung et al. indicate unelevated levels of IP10 in acute IgAV. However, in this study, 3 groups of patients were compared (with IgAV, Kawasaki disease, and acute febrile infectious disease) without comparison with healthy control [46].

The factors limiting the value of this study are the retrospective character of single-center studies, a small study group, different time from the onset of the disease symptoms to the collection of a blood sample and no cytokine determinations in the convalescent phase.

In conclusion, all tested proteins stimulate neutrophils and other immune cells migration, adhesion to the vascular endothelium, and their infiltration into the perivascular space, which results in the development of leukocytoclastic vasculitis. Our results showed that although the serum IL-17A, IL-18, and IL-23 levels increase in the acute phase of IgAV, there is no significant correlation between them and clinical symptoms, regardless of how they were evaluated, whether by systemic involvement, or whether by the objective clinical scoring scale of the disease severity. Currently, they cannot be used as indicators for predicting the course of the disease. Among the tested cytokines, IL-17A seems to be a good predictor of IgAV occurrence. It is important to conduct further studies on the parameters that are useful for predicting the severity of the disease and selection of the patients who require increased supervision. Searching for new indicators to facilitate clinical decision making is needed as well.
